# Dietary fat stimulates development of NAFLD more potently than dietary fructose in Sprague–Dawley rats

**DOI:** 10.1186/s13098-018-0307-8

**Published:** 2018-01-24

**Authors:** Victoria Svop Jensen, Henning Hvid, Jesper Damgaard, Helle Nygaard, Camilla Ingvorsen, Erik Max Wulff, Jens Lykkesfeldt, Christian Fledelius

**Affiliations:** 1 Department of Veterinary and Animal Science, Faculty of Health and Medical Sciences, University of Copenhagen, Ridebanevej 9, 1870 Frederiksberg, Denmark; 2 Insulin Pharmacology, Novo Nordisk A/S, Novo Nordisk Park 1, 2760 Maaloev, Denmark; 3 Histology and Imaging, Novo Nordisk A/S, Novo Nordisk Park 1, 2760 Maaloev, Denmark; 4 Obesity and Diabetes Pharmacology, Novo Nordisk A/S, Novo Nordisk Park 1, 2760 Maaloev, Denmark

**Keywords:** Non-alcoholic fatty liver disease, NAFLD, Animal models, Diet, Fat, Fructose, Cholesterol, Non-alcoholic steatohepatitis, NASH, Rat

## Abstract

**Background:**

In humans and animal models, excessive intake of dietary fat, fructose and cholesterol has been linked to the development of non-alcoholic fatty liver disease (NAFLD). However, the individual roles of the dietary components remain unclear. To investigate this further, we compared the effects of a high-fat diet, a high-fructose diet and a combination diet with added cholesterol on the development of NAFLD in rats.

**Methods:**

Forty male Sprague–Dawley rats were randomized into four groups receiving either a control-diet (Control: 10% fat); a high-fat diet (HFD: 60% fat, 20% carbohydrate), a high-fructose diet [HFr: 10% fat, 70% carbohydrate (mainly fructose)] or a high-fat/high-fructose/high-cholesterol-diet (NASH: 40% fat, 40% carbohydrate (mainly fructose), 2% cholesterol) for 16 weeks.

**Results:**

After 16 weeks, liver histology revealed extensive steatosis and inflammation in both NASH- and HFD-fed rats, while hepatic changes in HFr-rats were much more subtle. These findings were corroborated by significantly elevated hepatic triglyceride content in both NASH- (*p* < 0.01) and HFD-fed rats (*p* < 0.0001), elevated hepatic cholesterol levels in NASH-fed rats (*p* < 0.0001), but no changes in HFr-fed rats, compared to Control. On the contrary, only HFr-fed rats developed dyslipidemia as characterized by higher levels of plasma triglycerides compared to all other groups (*p* < 0.0001). Hepatic dysfunction and inflammation was confirmed in HFD-fed rats by elevated levels of hepatic MCP-1 (*p* < 0.0001), TNF-alpha (*p* < 0.001) and plasma β-hydroxybutyrate (*p* < 0.0001), and in NASH-fed rats by elevated levels of hepatic MCP-1 (*p* < 0.01), increased hepatic macrophage infiltration (*p* < 0.001), and higher plasma levels of alanine aminotransferase (*p* < 0.0001) aspartate aminotransferase (*p* < 0.05), haptoglobin (*p* < 0.001) and TIMP-1 (*p* < 0.01) compared to Control.

**Conclusion:**

These findings show that dietary fat and cholesterol are the primary drivers of NAFLD development and progression in rats, while fructose mostly exerts its effect on the circulating lipid pool.

## Background

Non-alcoholic fatty liver disease (NAFLD) constitutes an increasingly prevalent liver disorder and has been suggested to be the hepatic manifestation of the metabolic syndrome [1]. At present, NAFLD is assuming epidemic proportions affecting more than 25% of the world’s population, likely related to a concurrent rise in the prevalence of obesity and type 2 diabetes [2], although an increasing proportion of normal weight individuals are also affected pointing towards dyslipidemia as an important independent risk factor [3]. NAFLD denotes a broad range of liver pathologies, spanning from simple hepatic steatosis, to advanced non-alcoholic steatohepatitis (NASH), which without intervention may progress to transplant-requiring cirrhosis and hepatocellular carcinoma. Whereas simple hepatic steatosis is mainly characterized by lipid accumulation in > 5% of hepatocytes, NASH is a condition further complicated by lobular inflammatory infiltrations and the presence of ballooning hepatocytes with or without concurrent fibrosis [4].

It has been hypothesized that progression through NAFLD stages involves multiple adverse “hits” where both hepatic inflammation and oxidative stress are key facilitators [5]. Accordingly, serum and hepatic levels of proinflammatory cytokines such as tumor-necrosis factor alpha (TNF-α) and monocyte-chemoattractant protein-1 (MCP-1) have been found to be increased in patients with both simple hepatic steatosis and NASH [6–9]. Also, the glycoproteins haptoglobin and tissue-inhibitor of metalloproteinase 1 (TIMP-1) have been shown to be elevated in plasma of patients with more advanced stages of NAFLD and have therefore recently been suggested as useful clinical plasma biomarkers, indicative of hepatocyte ballooning and liver fibrosis, respectively [10, 11].

Even though the specific etiology of NAFLD remains unclear, dietary fat and cholesterol have been linked to the development of hepatic steatosis and NASH in both humans and animal models [12–14] and more recently, the marked increase in consumption of the simple carbohydrate fructose, has been pointed out as another possible contributory factor [15, 16]. The realization that diet is an important contributor to the pathogenesis of NAFLD has resulted in a considerable variety of diet-induced animal models, where the majority (apart from those based on nutritional deficiencies) is based on a high-fat diet with varying levels of simple carbohydrate and cholesterol [17, 18]. However, the individual roles of fat, carbohydrates and cholesterol in the development of NAFLD are still not entirely clear. Thus, improved insight from animal studies on the individual contribution of fat, simple carbohydrates and cholesterol to the metabolic and inflammatory characteristics of NAFLD is an important prerequisite in understanding the disease complexity in patients. Furthermore, establishment of animal models of NAFLD/NASH that more adequately mimic human pathology will be valuable when testing novel therapeutics for NAFLD/NASH.

The aim of the present study was therefore to compare the effects of diets high in either dietary fat, dietary fructose or a combination diet with added cholesterol on development of NAFLD, dyslipidemia and inflammation in Sprague–Dawley rats. Development of NAFLD was evaluated over time both histologically and biochemically and quantitative computed tomography (qCT) was used as a non-invasive marker of hepatic steatosis, enabling monitoring of NAFLD progression throughout the study period.

## Methods

### Animals

Forty male Sprague–Dawley rats were purchased from Charles River Laboratories (Sulzfeld, Germany). Animals were acclimatized for 2 weeks upon arrival and were 12 weeks old at the beginning of the experiment, weighing approximately 440–460 g. They were housed two per cage with access to non-chlorinated, non-acidific tap-water and with unrestricted access to standard rodent chow (Altromin 1324, Brogaarden, Denmark) until initiation of the experiment. Temperature in the animal rooms was maintained at 20–25 °C with a light/dark cycle of 12/12 h, a relative humidity of 30–70%, and air change 8–15 times/h.

### Experimental design

Immediately prior to study start, rats were block-randomized into four diet groups based on body weight (n = 10/group). Briefly, rats were sorted in order of ascending weight into blocks, and within each block, rats were then randomly assigned to receive either a control diet (Control), a high-fat diet (HFD), a high-fructose diet (HFr) or a high-fat/high-fructose/high-cholesterol-diet (NASH), (Research Diets, NJ, USA) for 16 weeks. The length of the study period was chosen to enhance the probability that NASH with concurrent fibrosis would be established, as has previously been described in Sprague–Dawley rats fed a high-fat diet [19]. The exact nutritional composition of each diet is shown in Table 1. All diets were stored at − 20 °C throughout the study until use, and feed-remains were exchanged with fresh feed twice weekly to prevent purification. To obtain baseline values for all parameters assessed at the 16 week time point, animals were qCT-scanned, quantitative magnetic resonance (qMR)-scanned and blood sampled at study start. After 16 weeks, animals were euthanized by exsanguination under isoflurane anesthesia by incision of the abdominal aorta. Immediately following exsanguination, the liver, a 5 cm section of the proximal jejunum and the two epididymal fat depots were excised, weighed and freeze clamped for further analysis. Food intake and body weight was measured twice weekly throughout the study. Food intake was recorded by calculating the difference between the amount of administered food and remaining food in the cage. These data were used to calculate the accumulated energy intake after the 16 weeks. qCT- and qMR-scans was performed on a bi-monthly basis, and blood samples were collected at baseline and at study termination.

**Table 1:** Nutritional composition of diets

Nutrient composition	Control	NASH	HFD	HFr
Fat (%)	10	40	60	10
Carbohydrates (total) (%)	70	40	20	70
Fructose (%)	0	20	0	60
Sucrose (%)	0	10	7	1
Corn starch (%)	55	0	0	9
Maltodextrin 10 (%)	15	10	13	0
Protein (%)	20	20	20	20
Cholesterol (%)	–	2	–	–
Metabolizable energy (kcal/g)	3.85	4.49	5.24	3.85

*NASH* NASH-diet, *HFD* high-fat diet, *HFr* high-fructose diet

### Plasma samples

Blood samples were taken from the sublingual vein in conscious non-fasted animals. They were collected in K_3_-EDTA microvettes and after centrifugation plasma was isolated and kept at − 20 °C until further analysis. Triglycerides (TG), total cholesterol (TC), high-density lipoprotein cholesterol (HDL-C), free fatty acids (FFA), alanine aminotransferase (ALAT), aspartate aminotransferase (ASAT), Haptoglobin and β-hydroxybutyrate were measured using a Cobas 6000 c501 instrument (Roche Diagnostics GmbH, D-68296 Mannheim, Germany), according to manufacturer’s instructions. Plasma levels of MCP-1 and TIMP-1 were analyzed using a multiplex assay, (K15179-C1, Mesoscale Discovery, MD, USA).

Additionally, plasma samples were collected from 4-h fasted animals at week 15 (collected 1 week prior to week 16 blood samples, to avoid the fasting interfering with plasma lipid parameters) and assayed for endogenous insulin and glucose. Samples for blood glucose measurements (10 µL) were collected in capillary tubes and transferred to 500 µL system solution. Blood glucose levels were analyzed using the glucose oxidase method at a Biosen apparatus (EKF Diagnostics, Barleben, Germany) according to manufacturer’s instructions. Samples for endogenous insulin measurements were collected in K_3_-EDTA microvettes and after centrifugation; plasma was isolated and analyzed as previously described [20]. Leptin levels were quantified using a Milliplex assay (RADPKMAG80-K, Merck, Hellerup, DK).

### qMR and qCT

To determine total fat mass, all animals underwent qMR-scans after 8 and 16 weeks using an EchoMRI Body Composition Analyser (EchoMRI, Houston, TX, USA). Mass measurements of fat tissue were performed according to manufacturer’s instructions and as previously described [21].

In order to assess the development and progression of NAFLD, qCT scans were used to quantify liver density as an indirect measure of hepatic fat content. Liver qCT-scans were performed after 8 and 16 weeks in isoflurane-anesthetized rats using a Latheta CT-scanner (LCT-200 series, Aloka co. LTD, Tokyo, Japan). Changes in liver density were calculated by subtracting baseline qCT values from week 8 and 16 time point values.

### Liver biochemistry

Levels of hepatic TG, TC and liver glycogen were analyzed on homogenized liver tissue sampled from the left lateral lobe using a Cobas 6000 c501 instrument (Roche Diagnostics GmbH 68206 Mannheim, Germany) according to manufacturer’s instructions and as previously described [22].

### Histology

The right medial, the left lateral, and the caudate lobe of the liver were collected for histological examination. Samples were fixed in 10% Neutral Buffered Formalin, processed to paraffin, imbedded and cut in 2–4 µm sections. In addition, a sample from the left medial lobe was snap frozen for cryo-sectioning. Steatosis was evaluated in all three lobes with Mayer’s Haematoxylin and Eosin (H&E) on paraffin section (10 animals/gr) and confirmed by Oil Red O-stain on frozen cryo-sections of the left medial lobe (2–3 animals/group). Inflammation and fibrosis were evaluated based on the morphology on the H&E stain, collagen deposition on a Picro Sirius stain and macrophage infiltration based on a CD68 immunohistochemistry (IHC) stain. For the CD68 IHC stain, antigens were first retrieved by TEG buffer pH 9.0 and subsequently blocked with 0.5% hydrogen peroxide followed by avidin and biotin (Invitrogen, CA, USA). The slides were then incubated with primary antibody (3 µg/mL, MCA341R, AbD serotec, CA, USA) for 60 min, biotinylated secondary antibody for 60 min (1 µg/mL, 715-065-151, Jackson ImmunoSearch, PA, USA) and ABC reagent (Vector Laboratories, CA, USA). Finally, macrophages were visualised with a DAB reagent (Dako, Glostrup, Denmark). All sections were scanned with a Hamamutsu slide scanner and later evaluated using NanoZoomer Digital Pathology Image software (Hamamatsu, Hamamatsu City, Japan). The collagen and macrophage areas were quantified by Visiomorph software (Visiopharm, Hoersholm, Denmark).

### Inflammatory markers in tissues

As described above, livers, epididymal fat depots, and jejunal segments were excised from the animals immediately after sacrifice and stored at − 80 °C until analysis. Tissue protein concentrations of the three tissues were first determined using a Pierce BCA Protein Assay Kit (Thermo Fisher Scientific, MA, USA) method according to manufacturer’s instruction. TNF-α and MCP-1 levels were subsequently determined in tissue homogenates with enzyme-linked immunosorbent assay (ELISA) kits (AB100785 and AB100778; Abcam, Cambridge, UK) according to manufacturer’s instructions. Absorbance was read using a Spectramax 340PC384 microplate reader at 450 nm (Molecular Devices, CA, USA).

### Statistical analysis

Statistical analyses were done using GraphPad Prism version 6.05 (GraphPad Software Inc., La Jolla, CA, USA). Data were assumed to be normally distributed and confirmed by visual inspection of qq-plots. In case of severe deviations from normality, statistical analyses were performed on log-transformed data (natural logarithm) or with the use of non-parametric tests. Data are presented as mean ± SEM, except for log-transformed data, which are presented as geometric means with 95% confidence intervals. Differences in means between diet groups for each parameter were analyzed using one-way ANOVA, repeated measures two-way ANOVA or Kruskal–Wallis tests where appropriate, and compared after 16 weeks on the diets. Bonferroni or Dunn corrections, respectively, were used to adjust for multiple comparisons. Outliers in data sets were identified and removed using the ROUT-function in GraphPad Prism. p-values < 0.05 were considered significant.

## Results

Baseline characteristics for Control, HFD, HFr and NASH-groups are given in Table 2 and show that groups were comparable at study start for all parameters.

**Table 2:** Baseline characteristics of rats immediately after randomization to either Control-, NASH-, HFD- or HFr-diets

	Control	NASH	HFD	HFr
Body weight (g)	454.9 ± 6.6	457.7 ± 5.7	442.7 ± 7.0	447.7 ± 8.8
Fat mass (g)	43.6 ± 2.6	44.4 ± 2.4	46.7 ± 2.0	44.9 ± 2.6
Liver density (HU)	40.0 ± 1.0	39.6 ± 0.6	40.7 ± 1.0	38.9 ± 0.8
Plasma TG (mM)	0.9 ± 0.1	0.9 ± 0.1	1.0 ± 0.1	1.3 ± 0.2
Plasma FFA (mM)	0.3 ± 0.02	0.3 ± 0.03	0.4 ± 0.03	0.3 ± 0.02
Plasma cholesterol (mM)	1.6 ± 0.1	1.5 ± 0.1	1.7 ± 0.1	1.6 ± 0.1
Plasma HDL-C (mM)	1.0 ± 0.04	1.0 ± 0.1	1.1 ± 0.04	1.0 ± 0.1
ALAT (U/L)	33.0 ± 2.1	31.9 ± 1.7	35.7 ± 2.8	33.1 ± 1.9
ASAT (U/L)	62.4 ± 1.9	59.9 ± 3.5	61.5 ± 2.9	63.2 ± 2.3

Results are presented as mean ± SEM

*HU* Hounsfield units, *TG* triglycerides, *FFA* free fatty acids, *HDL-C* high-density-lipoprotein-cholesterol, *ALAT* alanine aminotransferase, *ASAT* aspartate aminotransferase. Sample sizes: n = 10/group

### Effects of diet on body weight, energy intake and fat distribution

**Fig. 1:**
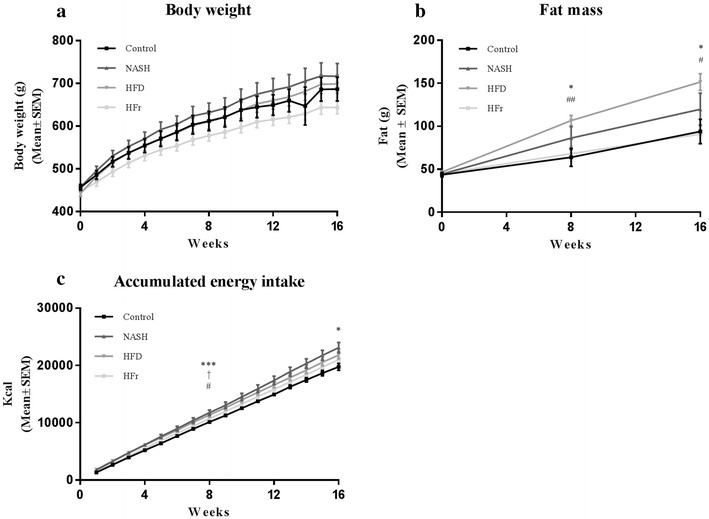
Effects on body weight, fat mass and energy intake of Control-, NASH-, HFD- and HFr-diets. **a** Body weight increased in all 4 diet groups throughout the study, but did not differ after 16 weeks. **b** After 8 weeks fat mass was significantly increased in HFD-fed rats compared to Control- and HFr-fed rats. This effect was still present at week 16. **c** After 8 weeks, accumulated energy intake was significantly higher in NASH-fed rats, compared to both Control- and HFr-fed rats and in HFD-fed rats compared to Control. This remained significant only in NASH-fed compared to Control-fed rats after 16 weeks. Comparisons between groups: *NASH vs. Control; ^#^NASH vs. HFr; ^†^HFD vs. Control. Statistical significance: *p < 0.05; ***p < 0.001; ^#^p < 0.05; ^##^p < 0.01; ^†^p < 0.05

Figure 1 shows changes in body weight, fat mass and energy intake. Body weight increased throughout the study in all groups, but did not differ from Control after 16 weeks (Fig. 1a). Fat mass was significantly increased in HFD-fed animals compared to both those fed Control and HFr-diets (*p* < 0.05, Fig. 1b) after 16 weeks. After 8 weeks, accumulated energy intake was significantly higher in NASH-fed rats, compared to both Control- and HFr-fed rats (*p* < 0.001 and *p* < 0.05, Fig. 1c) and in HFD-fed rats compared to Control (*p* < 0.05*)*. This difference remained significant only in NASH-fed compared to Control-fed rats after 16 weeks (*p* < 0.05).

### Effects of diet on the liver

**Fig. 2:**
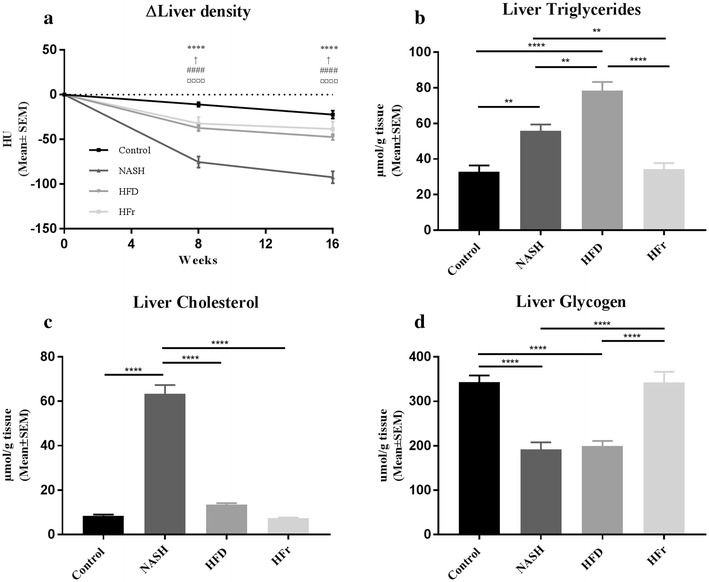
Effects on liver in Control-, NASH-, HFD- and HFr-fed rats. **a** Throughout the study, liver density continuously declined in all four diet groups, with the effect being more pronounced in the NASH-group. Both halfway through the study period and at the week 16 time point the decline in liver density was significantly greater in NASH and HFD compared to Control and in NASH compared to HFD and HFr. **b** Liver triglyceride content was significantly elevated in NASH- and HFD-fed rats compared to Control and HFr. Additionally, HFD-fed rats had significantly higher hepatic TG levels compared to NASH-fed. Hepatic TG levels in rats fed HFr did not differ from those fed the Control-diet. **c** Hepatic cholesterol levels were significantly elevated in NASH-fed animals compared to Control, HFD, and HFr. **d** The level of liver glycogen was significantly lower in both NASH and HFD compared to Control and HFr. **a** Comparisons between groups: *Control vs. NASH; ^†^Control vs. HFD; ^¤^NASH vs. HFD; ^#^NASH vs. HFr. Statistical significance: ****p < 0.0001; ^†^*p* < 0.05; ^¤¤¤¤^*p* < 0.0001; ^####^*p* < 0.0001. **b**–**d**. Statistical significance: **p < 0.01; ****p < 0.0001. *TG* triglyceride, *TC* total cholesterol, *NASH* NASH-diet, *HFD* high-fat diet, *HFr* high fructose diet. Results are shown as mean ± SEM

Throughout the study, liver density (used as an indirect measure of liver fat content) continuously declined in all four diet groups as measured by qCT with the effect being most pronounced in the NASH-group (Fig. 2a). At both 8 and 16 weeks, the decrease in liver density was significantly greater in NASH and HFD compared to Control (*p* < 0.0001 and *p* < 0.05) and in NASH compared to HFD and HFr (*p* < 0.0001).

**Fig. 3:**
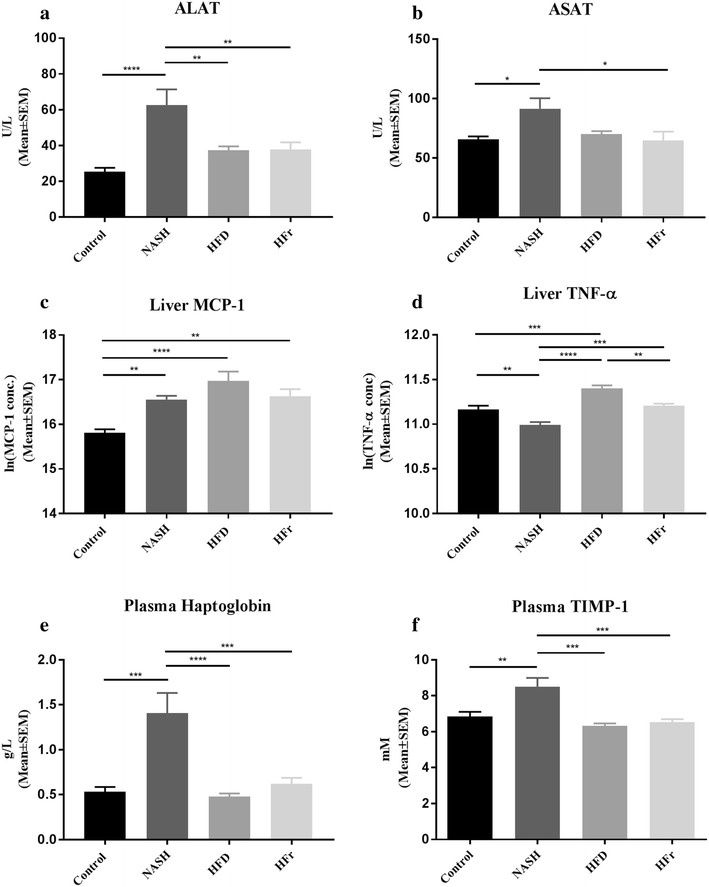
Markers of hepatic function, inflammation and fibrosis. **a** After 16 weeks ALAT-levels were significantly higher in NASH-fed rats compared to Control-, HFD- and HFr-fed rats. **b** Plasma levels of ASAT were higher in the NASH-group compared to both Control and HFr-groups. **c** Rats on NASH, HFD and HFr-diet had increased hepatic levels of MCP-1 compared to Control. **d** Hepatic TNF-α levels were significantly increased in the HFD fed rats compared to Control. **e** Haptoglobin was significantly increased in plasma of NASH-fed rats compared to rats fed Control, HFD and HFr. **f** Plasma TIMP-1 was significantly increased in the NASH-fed rats, compared to all other groups. Statistical significance: *p < 0.05; **p < 0.01; ***p < 0.001; ****p < 0.0001. Results are shown as mean ± SEM

Livers from NASH-fed rats weighed significantly more compared to Control (*p* < 0.0001), HFD (*p* < 0.0001) and HFr (*p* < 0.001, Table 3), as did livers from HFr-fed rats compared to HFD (*p* < 0.05). Hepatic TG content was higher in NASH- and HFD-fed rats compared to both Control (*p* < 0.01 and *p* < 0.0001) and HFr (*p* < 0.01 and *p* < 0.0001, Fig. 2b). Additionally, HFD-fed animals had higher hepatic TG levels compared to NASH (*p* < 0.01). Hepatic TG levels in rats fed HFr did not differ from those fed the Control diet. Hepatic TC levels were significantly elevated in NASH-fed animals compared to Control, HFD, and HFr (*p* < 0.0001, Fig. 2c). Liver glycogen was significantly lower in both NASH and HFD compared to Control and HFr (*p* < 0.0001, Fig. 2d). At week 16, ALAT-levels were significantly higher in NASH-fed rats compared to Control- (*p* < 0.0001, Fig. 3a), HFD- and HFr-fed rats (*p* < 0.01). Plasma levels of ASAT were higher in the NASH-group compared to both Control- and HFr-groups (*p* < 0.05, Fig. 3b). Furthermore, plasma levels of the β-oxidation marker β-hydroxybuturate were increased in HFD-fed rats compared to Control-, NASH- and HFr-groups (*p* < 0.0001, *p* < 0.01 and *p* < 0.0001, Table 3).

**Table 3:** Metabolic and inflammatory effects in rats after 16 weeks on NASH-, HFD- and HFr-diet

	Control	NASH	HFD	HFr
Liver weight (g)	18.9 ± 3.4^#^	32.7 ± 7.9*^†‡^	16.5 ± 2.5^# ‡^	23.1 ± 4.4^#†^
Fasting plasma glucose (mM)^b^	6.4 ± 0.2^#†‡^	7.2 ± 0.2*	7.5 ± 0.1*	7.16 ± 0.2*
Fasting plasma insulin (pM)^b^	560.6 ± 79.5	486.7 ± 93.7	603.0 ± 43.7	695.8 ± 83.7
Plasma β-hydroxybutyrate (µmol/L)	38.4 ± 4.2^†^	64.0 ± 5.3^†^	115.4 ± 16.8*^#‡^	38.4 ± 2.8^†^
Plasma leptin (ng/mL)	3283.3 ± 1035.2^†^	3803.0 ± 1341.1^†^	9366.6 ± 2052.8*^#‡^	2638.0 ± 808.5^†^
Plasma TG (mmol/L)	1.6 ± 0.2^‡^	1.7 ± 0.1^‡^	1.5 ± 0.1^‡^	3.9 ± 0.5*^#†^
Plasma FFA (mmol/L)	0.3 ± 0.1	0.4 ± 0.03	0.4 ± 0.02	0.3 ± 0.02
Plasma Cholesterol (mmol/L)	2.1 ± 0.1	2.5 ± 0.1	2.1 ± 0.1	2.3 ± 0.2
Plasma HDL-C (mmol/L)	1.3 ± 0.1	1.0 ± 0.1	1.3 ± 0.1	1.1 ± 0.1
Jejunal MCP-1 (ng/mL)^a^	45.3 [36.3; 55.3]	58.1 [48.5; 69.6]	49.5 [40.1; 60.5]	56.4 [48.1; 65.5]
Jejunal TNF-α (ng/mL)^a^	179.9 [129.3; 247.7]	87.6 [61.7; 125.5]	171.1 [76.1; 384.6]	213.2 [112.4; 404.3]
Adipose tissue MCP-1 (ng/mL)^a^	14.8 [13.4; 16.5]	14.1 [11.9; 16.8]^‡^	14.2 [11.3; 17.7]^‡^	25.6 [15.2; 42.6]^#†^
Adipose tissue TNF-α (ng/mL)^a^	15.4 [14.4; 16.4]	16.1 [14.3; 18.2]	15.4 [13.6; 18.2]	16.2 [14.6; 18.0]

*TG* triglycerides, *FFA* free fatty acids, *HDL-C* high-density-lipoprotein-cholesterol, *MCP-1* monocyte-chemoattractant-protein-1, *TNF-α* tissue-necrosis-factor-alpha

Results are presented as mean ± SEM. Metabolic parameters denoted with superscript ^a^, were analysed on log-transformed data (due to non-normal distribution), and are consequently presented as geometric means with 95% confidence intervals. Superscripts (*,^#^,^†^,^‡^) indicate significant (*p* < 0.05) differences in readouts compared to Control-, NASH-, HFD- and HFr-groups respectively. Sample sizes at week 16: n = 6–10/group. ^b^ Fasting plasma glucose and fasting plasma insulin levels were assessed after 15 weeks

**Fig. 4:**
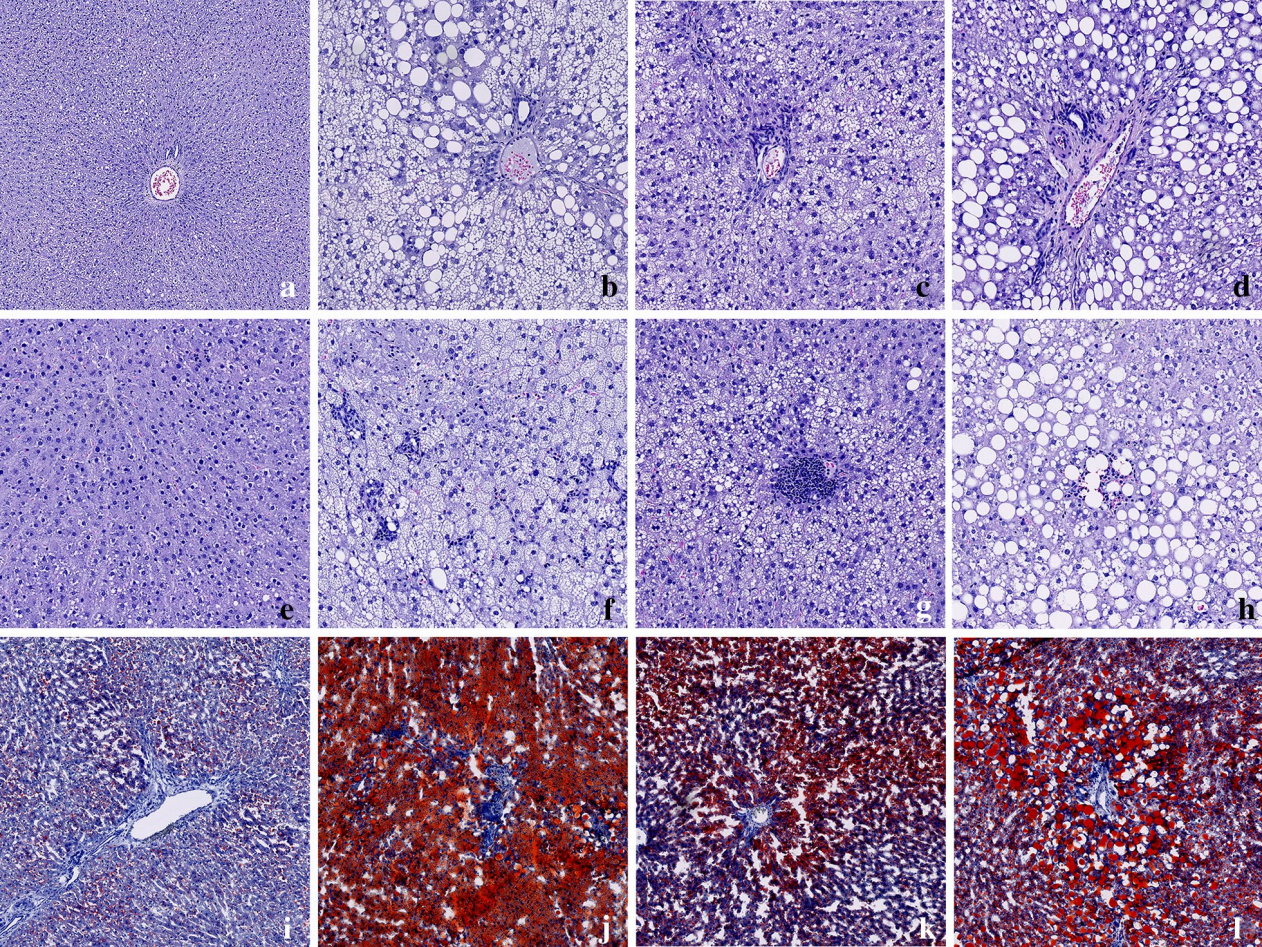
Histological evaluation of liver sections from Control-, NASH-, HFD- and HFr-fed rats. Row 1: representative H&E-stains of normal liver from **a** Control-fed rat, and hepatic steatosis in **b** NASH-fed rat, **c** HFD-fed rat and **d** HFr-fed rat. Hepatic steatosis in HFD-fed rats was almost exclusively found to be microvesicular (**c**); in HFr-fed rats almost exclusively macrovesicular (**d**); while steatosis in NASH-fed rats represented an intermediate between the two, with both macro- and microvesicular steatosis (**b**). Row 2: higher magnification of representative H&E stained liver sections. **e** Liver morphology appeared normal in Control-fed rats, whereas inflammatory infiltrates were observed in livers from **f** NASH-fed rats, **g** HFD-fed rats and **h** HFr-fed rats Row 3: Oil Red O stains of liver sections from **j** NASH-fed rats, **k** HFD-fed rats and **l** HFr-fed rats confirmed hepatic steatosis observed in **b**–**d**. **i** Oil Red O staining of liver from Control-fed rat

**Fig. 5:**
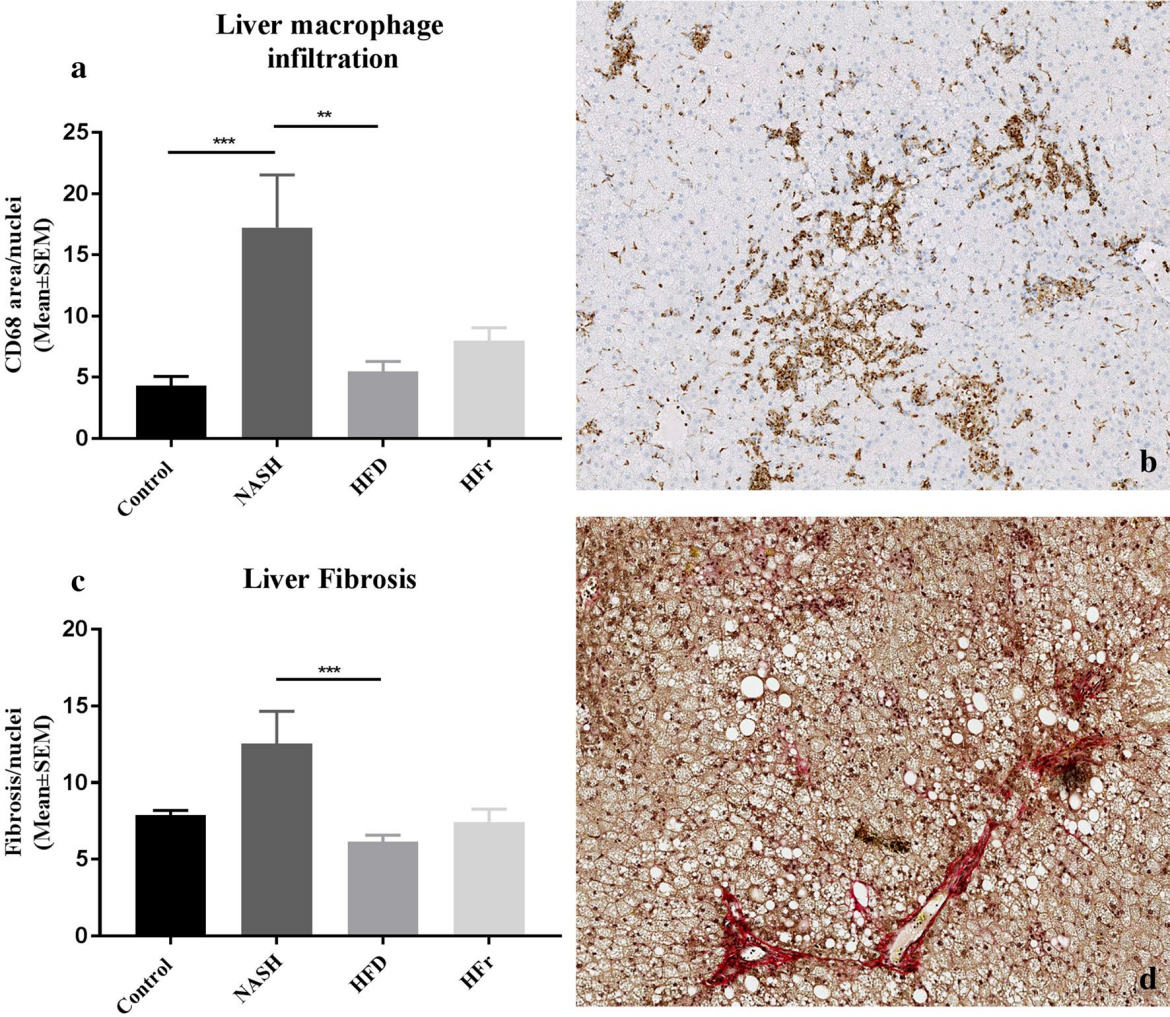
Macrophage infiltration and collagen deposition. A significant increase in macrophage infiltration was seen in NASH-fed rats, compared to Control- and HFD.fed rats (**a**) and collagen deposition in NASH-fed rats trended towards being increased, although only significantly when compared to HFD-rats (**b**). Representative CD68- (**c**) and Picro Weigert-stains (**d**) of liver from NASH-fed rat. Statistical significance: **p < 0.01; ***p < 0.001. Results in **a** and **c** are shown as mean ± SEM

The histological evaluation is presented in Fig. 4. After 16 weeks, hepatic steatosis was present in NASH, HFD and HFr-fed rats as confirmed by corresponding Oil Red O stains. No pathological changes were observed in livers of Control-fed rats. Steatosis was phenotypically distinguishable between NASH-, HFD- and HFr-fed rats. While zonal distribution was comparable (originating in zone 1, periportally), steatosis in HFD-rats was almost exclusively found to be of the microvesicular type; in HFr-fed rats almost exclusively macrovesicular; while the NASH-fed rats represented an intermediate between the two, with both macro- and microvesicular steatosis. Furthermore, steatosis in the NASH-group at week 16 was much more pronounced, involving not only zone 1, but occasionally also zones 2 and 3 and in some animals, only few areas displayed normal liver architecture. Inflammatory hepatic infiltration was identified in both NASH-, HFD- and HFr-fed rats. In all three groups, inflammation was characterized by being of primarily mononuclear composition with occasional neutrophilic involvement. However in HFr-fed rats, inflammatory cells appeared to center around lipid-loaded hepatocytes and form structures resembling lipogranulomas, whereas inflammatory infiltrates observed in HFD-fed rats were randomly scattered and not consistently associated with steatosis. As with the steatosis, inflammatory changes in the NASH-group were more pronounced, as exemplified by larger and more disseminated infiltrates. Accordingly, hepatic macrophage numbers were two- to threefold increased in the NASH-group compared to Control- and HFD-fed rats (*p* < 0.001 and *p* < 0.01, Fig. 5a, b). Hepatic deposition levels of collagen was higher in NASH-animals than in other groups, albeit only significantly increased compared to HFD (*p* < 0.001, Fig. 5c, d).

### Effects of diet on plasma lipids and general metabolic state

Plasma FFA did not differ between groups (Table 3). HFr-feeding induced a significant increase in circulating TG, compared to Control-, NASH- and HFD (*p* < 0.0001, Table 3). Plasma HDL-c levels were similar between groups, as were the plasma cholesterol levels (Table 3). Fasting hyperglycemia was present in all groups compared to Control after 16 weeks (NASH and HFr: *p* < 0.01, HFD: *p* < 0.0001, Table 3). However, fasting plasma insulin levels did not differ between groups (Table 3). Circulating leptin levels were significantly increased in the HFD-fed rats compared to both Control- (*p* < 0.05), NASH- (*p* < 0.05) and HFr-fed rats (*p* < 0.01, Table 3).

### Effects of diet on markers of systemic and tissue-specific inflammation

Hepatic levels of MCP-1 were significantly increased in rats on NASH (*p* < 0.01), HFD diet (*p* < 0.0001) and HFr diet (*p* < 0.01) compared to Control (Fig. 3c). Additionally, hepatic TNF-α levels were higher in HFD fed rats compared to Control- (*p* < 0.001), NASH (*p* < 0.0001) and HFr (*p* < 0.01, Fig. 3d). Haptoglobin was significantly increased in plasma of NASH-fed rats compared to rats fed Control (*p* < 0.001), HFD (*p* < 0.0001) and HFr (*p* < 0.001, Fig. 3e). Concentrations of MCP-1 and TNF-alpha were not elevated in adipose or intestinal tissue in any of the diet-groups compared to Control; however, HFr-fed rats had significantly higher levels of adipose tissue MCP-1 compared to both NASH- and HFD-fed rats (*p* < 0.05, Table 3). MCP-1 was below detection limit in plasma in all groups. Plasma TIMP-1 levels was significantly increased in the NASH-fed rats, compared to all other groups (vs. Control: *p* < 0.01; vs. HFD and HFr: *p* < 0.001, Fig. 3f).

## Discussion

The present study shows that feeding rats diets high in either dietary fat or dietary fructose results in distinctly different hepatic, metabolic and inflammatory profiles in rats. When comparing the effect of dietary fat and fructose, high-fat feeding more potently induce development of fatty liver and associated hepatic inflammation without affecting the circulating lipid pool. In contrast, high-fructose feeding appear to have the most pronounced effects on the plasma lipid profile, while only subtle effects on the liver are observed. The combination of fat, fructose, and cholesterol exacerbated and intensified overall effects on the liver. The strength of this study is the direct comparison of dietary fat (with very restricted amounts of carbohydrate) and dietary fructose (with very restricted amounts of fat) on parameters associated with NAFLD. This enables a more detailed evaluation of the individual roles of these macronutrients in disease progression. Furthermore, the use of qCT allows the non-invasive assessment of NAFLD-progression throughout the study, a method which to our knowledge has not previously been applied in rat studies comparing NAFLD progression after administration of different diets.

NAFLD in humans is often associated with obesity and insulin resistance, and these metabolic derangements were more closely reflected in the HFD-group. Accordingly, only HFD-fed rats became obese and even though the cumulative energy intake in this group was transiently higher than Control animals at week 8, differences in energy intake did no longer account for the HFD-induced obesity after 16 weeks. Circulating leptin levels were also significantly elevated only in HFD-fed rats, reflecting the increase in adipose tissue depot size within this group. Fasting hyperglycemia was present in all groups at study termination, indicating disturbances in glucose metabolism, even though fasting insulin levels remained similar between groups. These disturbances were further corroborated by the increased levels of hepatic TG and decreased/unaltered hepatic glycogen observed in both NASH-, HFD- and HFr-fed rats, indicative of selective insulin resistance [23].

Hepatic steatosis was rapidly induced in both NASH- and HFD-fed rats and was shown using qCT to progress in a time-dependent manner. In line with this, higher levels of plasma β-hydroxybutyrate were observed in HFD-fed rats, suggestive of increased β-oxidation due to hepatic lipid overload. The role of hepatic β-oxidation in the pathogenesis of NAFLD remains controversial, but several studies in human NASH-patients have found serum levels of β-hydroxybutyrate to be increased [24, 25]. Although not significant, there was a trend towards an increase of this marker in NASH-fed rats as well. The development of hepatic steatosis in NASH- and HFD-fed rats agrees well with recent studies in other rodent species suggesting fat and cholesterol to be important drivers in the development of experimental NAFLD/NASH [14, 22]. The addition of dietary cholesterol (NASH-diet) caused the most detrimental liver changes, evident by significant accumulation of both hepatic TG and cholesterol, pronounced decreases in liver density, and severe morphological alterations in liver histology. In contrast to what was observed in HFD- and HFr-fed rats, these changes were also accompanied by increased plasma levels of ALAT and ASAT, suggesting impaired liver function. It has previously been observed in both mice [26, 27] and humans [28] that the combined intake of dietary fat and carbohydrates, or of fat and cholesterol [29] can result in accelerated and more severe effects in the liver. The majority of these changes could be driven by the amount of cholesterol in the NASH-diet (2%), as hepatic levels of cholesterol measured in NASH-fed rats were very high. Supporting this, two previous studies in mice have suggested that dietary cholesterol is important in facilitating progression from simple steatosis to NASH [30] and that free cholesterol loading can sensitize the liver to cytokine-mediated hepatocellular death, inflammation and oxidative stress [31]. This could account for the more aggravated inflammatory histological response, supported by the significant increase in infiltrating macrophages observed in livers of NASH-fed rats compared to both HFD and Control groups. Further studies are needed to determine if these changes are caused exclusively by the cholesterol content, by a synergistic effect of fat and fructose, or by a combination of all.

While fat and cholesterol both seem to contribute to the development of NAFLD, previous studies in rats have shown that also high levels of dietary fructose are capable of inducing hepatic steatosis, inflammation and increase oxidative stress markers within a relatively short time-span of only 2–5 weeks [32–34]. These are in contrast to our findings with the HFr-diet, which only induced subtle changes in the liver. While steatosis was observed histologically in some animals in the HFr-group the condition was not confirmed by increased levels of liver TG at the week 16 time point. The apparent discrepancy between histological and biochemical analyses within the HFr-group could result from sampling variation, a problem also commonly encountered in biopsy-guided diagnosis of human NAFLD/NASH [35]. Biochemical analyses of hepatic TG-content was performed on liver tissue sampled from the left lateral lobe and histologic evaluation of livers in the HFr-group (performed on both left lateral, right medial and the caudate lobe) did in fact, in some animals, show specific lobes to be more severely affected than others, indicating that steatosis, at least in rats fed fructose-enriched diets, may not be homogenously distributed throughout the liver. Further studies, confirming the heterogeneously distributed steatosis would be interesting in terms of evaluating and comparing histology, biochemistry and imaging of the liver in rodent models of NAFLD/NASH.

Dyslipidemia is one of the hallmarks of the metabolic syndrome and has been shown to be strongly associated with NAFLD [36]. Dyslipidemia associated with NAFLD is typically characterized by elevated levels of circulating TG and low-density-lipoprotein cholesterol (LDL-C), as well as decreased HDL-C levels [37]. Only rats fed HFr-diet developed dyslipidemia, as defined by the presence of hypertriglyceridemia. Previous studies have also found that dietary fructose potently increase TG in plasma within a relatively short timeframe in both rats and mice [34, 38]. In rats, this has been suggested to be caused in part by the ability of fructose to both increase hepatic very-low-density-lipoprotein (VLDL)-TG secretion and decrease VLDL-TG clearance from the circulation, even in the absence of hyperinsulinemia [39, 40]. Mechanistically, the increase in VLDL-TG secretion has been proposed to result from a combined effect of fructose-induced hepatic stress responses [40], and activation of hepatic enzymes involved in the de novo synthesis of fatty acids (de novo lipogenesis) [41].

In contrast to the HFr-diet, neither the HFD- nor the NASH-diet resulted in increased plasma TG, even though high-fat diets have previously been shown to induce hypertriglyceridemia in rats [42]. However, our findings agree with a recent study in mice investigating the contribution of dietary fat and cholesterol to NAFLD-development, which showed no effect of dietary fat and cholesterol on circulating TG or FFA levels, independently of whether these macronutrients were fed separately or in combination [29]. One explanation could be that accelerated fat accumulation in the liver imposes a significant strain on metabolic pathways responsible for the release of lipids from the liver into plasma. Accordingly, it has been shown both in vitro and in vivo that high levels of fat and cholesterol in the liver result in increased stress in the endoplasmic reticulum, limiting secretion of apolipoprotein B100 (an essential component in the assembly of VLDL-particles) and thereby inhibits hepatic export of TG [29, 43]. The inflammatory profile was clearly distinguishable between groups in the present study. Systemic inflammation was present only in NASH-fed rats with higher circulating levels of haptoglobin. We also found increased levels of the pro-inflammatory cytokines MCP-1 and TNF-alpha in liver tissue homogenates in NASH-, HFD- and HFr- and in HFD-fed rats, respectively, suggesting progression of hepatic steatosis towards NASH with the increased inability of the liver to cope with fat infiltration. The inflammatory infiltrates observed histologically in livers of HFr- and NASH-fed rats at week 16, were reflected only in significantly higher levels of hepatic MCP-1, not TNF-alpha. Compared to the other diet-groups, NASH-fed rats might be expected to have the highest levels of liver inflammatory cytokines, due to the higher macrophage count, increased liver enzymes, and the more severe liver pathology. However, hepatic macrophages are a remarkably heterogeneous population of immune cells, whose effector function depends on origin, underlying pathogenesis and disease stage, which can result in high diversity in inflammatory cytokines released and cell surface markers [44, 45]. This could explain why the NASH-fed group does not have the highest hepatic MCP-1 and TNF-alpha levels despite the higher macrophage count and the more severe liver pathology. Despite increased hepatic MCP-1 levels in all groups compared to Control, MCP-1 plasma levels were below detection limit.

It has been hypothesized that fructose and fat may induce their inflammatory action in the liver not only by dietary overload, but also by stimulation of bacterial overgrowth in the intestine, increasing intestinal permeability and thereby facilitating translocation of endotoxins across the intestinal barrier that are then transported to the liver [46, 47]. In the present study, we were not able to detect increased intestinal levels of TNF-alpha or MCP-1 in any diet group. Moreover, we did not find increased levels of MCP-1 and TNF-alpha in visceral adipose tissue in any of the groups, even though inflammation particularly in the adipose tissue compartment is strongly associated with NAFLD/NASH in humans [48]. Notably, the cytokine analyses in this study were performed on epididymal fat depots, which may not adequately represent visceral adipose tissue depots in humans [49].

Although fibrosis is not a prerequisite for the NASH diagnosis, it often accompanies the three recognized diagnostic hallmarks (steatosis, lobular inflammation and hepatocyte ballooning), and can be used as a grading tool for evaluation of NASH severity [50]. In the present study, neither cellular ballooning nor fibrosis was present in any of the groups. However, increased levels of plasma haptoglobin and TIMP-1 were observed in NASH-fed rats and are considered predictors of these pathological liver changes, indicating that the NASH diet might be promising in terms of modelling more advanced stages of NASH within a relatively short time-frame. The tendency towards increased collagen deposition in NASH-fed rats further supports this. The inability of the diets used in this study to induce liver fibrosis in rats after only 16 weeks is not entirely surprising. Successful dietary induction of fibrosis in rodent models of NASH has so far mainly been associated with either genetic manipulation, forced overfeeding or diets low in methionine and choline, [51, 52], except for the guinea pig that has been shown to develop NASH after 16 weeks of high fat feeding [22]. However, mild hepatic fibrosis in addition to a NASH-like phenotype has also been induced in C57BL/6 mice on a high-fat diet, but only after 50 weeks [53]. In a recent study, using levels of dietary cholesterol comparable to the level in the NASH-diet in our study, development of fibrosis in Sprague–Dawley rats was observed after only 9 weeks. [54]. However, the diets used by Ichimura and colleagues included relatively high levels of dietary cholate (2%), which is known to potently induce hepatotoxicity and upregulate specific genes related to hepatic fibrosis [55].

## Conclusion

Dietary fat seems to primarily drive NAFLD development in Sprague–Dawley rats with potent effects on hepatic fat accumulation and inflammation, whereas dietary fructose primarily affects circulating lipids with much more subtle effects on the liver. Combining fat, fructose and cholesterol accelerates NAFLD development and increase the overall severity of changes observed in the liver.

## Abbreviations

ALAT: alanine aminotransferase
ASAT: aspartate aminotransferase
ELISA: enzyme-linked immunosorbent assay
FFA: free fatty acid
H&E: Haematoxylin and Eosin
HDL-C: high-density lipoprotein cholesterol
HFD: high-fat diet
HFr: high-fructose diet
HU: Hounsfield unit
IHC: immunohistochemistry
LDL-C: low-density lipoprotein cholesterol
MCP-1: monocyte-chemoattractant-protein 1
NAFLD: non-alcoholic fatty liver disease
NASH: non-alcoholic steatohepatitis
qCT: quantitative computed tomography
qMR: quantitative magnetic resonance
TC: total cholesterol
TG: triglyceride
TIMP-1: tissue-inhibitor of metalloproteinase-1
TNF-α: tumor-necrosis-factor alpha
VLDL: very-low-density lipoprotein
